# Supplementary material for: Impact of Sertraline, Fluoxetine, and Escitalopram on Psychological Distress among United States Adult Outpatients with a Major Depressive Disorder
Source: Healthcare (Basel). 2023 Mar 3;11(5):740. doi: 10.3390/healthcare11050740 (PMC10001334; doi:10.3390/healthcare11050740)
Supplement: Supplementary file 1 [file healthcare-11-00740-s001.zip › healthcare-2223155-SI.pdf]

S1: Kessler Index (K6) score

The Kessler Index (K6) scores measure individuals' non-specific psychological distress in the past 30 days [28]. The scale consists of six items, each rated on a five-point Likert scale (from "none of the time" to "all of the time") [28]. The six items are:

1. "During the past 30 days, how often did you feel nervous?"
2. "During the past 30 days, how often did you feel hopeless?"
3. "During the past 30 days, how often did you feel restless or fidgety?"
4. "During the past 30 days, how often did you feel so depressed that nothing could cheer you up?”
5. "During the past 30 days, how often did you feel that everything was an effort?"
6. "During the past 30 days, how often did you feel worthless?" [28]
